# Supplementary material for: Identification of distinct pH- and zeaxanthin-dependent quenching in LHCSR3 from Chlamydomonas reinhardtii
Source: eLife. 2021 Jan 15;10:e60383. doi: 10.7554/eLife.60383 (PMC7864637; doi:10.7554/eLife.60383)
Supplement: Figure 1—source data 1. — The median (med) and standard deviation (std) are given for each sample. The 95% confidence intervals for each value were determined by bootstrapping the samples 10,000 times. [file elife-60383-fig1-data1.docx]

|  | **Med. Intensity (cp 10 ms)** | **Std. intensity (cp 10 ms)** |
| --- | --- | --- |
| **WT Vio pH 7.5** | 21.3 ± 1.5 | 12.8 ± 0.8 |
| **WT Vio pH 5** | 18.1 ± 1.4 | 14.3 ± 0.7 |
| **Stop Vio pH 7.5** | 18.5 ± 0.8 | 11.1 ± 0.8 |
| **Stop Vio pH 5** | 23.1 ± 1.6 | 18.4 ± 2.7 |
| **WT Zea pH 7.5** | 9.9 ± 0.5 | 9.5 ± 2.8 |
| **WT Zea pH 5** | 11.6 ± 0.8 | 9.3 ± 1.1 |
| **Stop Zea pH 7.5** | 12.4 ± 1.0 | 11.3 ± 0.9 |
| **Stop Zea pH 5** | 13.6 ± 0.8 | 13.2 ± 1.7 |
