# Supplementary material for: Identification of distinct pH- and zeaxanthin-dependent quenching in LHCSR3 from Chlamydomonas reinhardtii
Source: eLife. 2021 Jan 15;10:e60383. doi: 10.7554/eLife.60383 (PMC7864637; doi:10.7554/eLife.60383)
Supplement: Figure 2—source data 1. — The number of dynamic components, fluorescence lifetime states, intensity of each lifetime state, population of each state, and transition rates between states were estimated by global fitting of the correlation functions as shown in Figure 2—figure supplement 1 using the model function described in the Materials and methods and the fluorescence lifetime distributions shown in Figure 2A–D. The fluorescence intensity is a relative intensity that is normalized by the total measurement time for each sample and scaled to set the maximum intensity to 1. The free-energy differences were calculated as described in the Materials and methods. [file elife-60383-fig2-data1.docx]

Component

## pH 7.5

Fluorescence

# WT Vio

Component

| 1 | 2 | 3 |
| --- | --- | --- |

## pH 5.0

Fluorescence

| 1 | 2 | 3 |
| --- | --- | --- |

| Lifetime state | 1 | 2 | 1 | 2 | 1 | 2 |
| --- | --- | --- | --- | --- | --- | --- |
| Lifetime | 0.71 | 2.45 | 0.71 | 2.45 | 0.71 | 2.45 |
| Intensity | 0.10 | 0.10 | 0.080 | 0.32 | 0.18 | 0.13 |

| Lifetime state | 1 | 2 | 1 | 2 | 1 | 2 |
| --- | --- | --- | --- | --- | --- | --- |
| Lifetime | 0.68 | 2.43 | 0.68 | 2.43 | 0.68 | 2.43 |
| Intensity | 0.083 | 0.049 | 0.074 | 0.21 | 0.0013 | 0.046 |

Transition rates Transition rates

| Lifetime state Final \ Initial | 1 | 2 | 1 | 2 | 1 | 2 |
| --- | --- | --- | --- | --- | --- | --- |
| 1 | 0.25 23 | | 0.14 | 850 | 0.045 | 0.0017 |
| 2 | 15 0.25 | | 230 | 0.23 | < 0.001 | 0.039 |
| Population | 0.61 | 0.39 | 0.79 | 0.21 | 0.94 | 0.060 |
| Free energy  diﬀerence | 90.0 | | 278 | | 587 | |

| Lifetime state Final \ Initial | 1 | 2 | 1 | 2 | 1 | 2 |
| --- | --- | --- | --- | --- | --- | --- |
| 1 | 0.27 23 | | 0.22 | 780 | 0.49 | 0.0022 |
| 2 | 5.1 0.32 | | 250 | 0.28 | < 0.001 | 0.10 |
| Population | 0.83 | 0.17 | 0.72 | 0.28 | 0.95 | 0.050 |
| Free energy  diﬀerence | 314 | | 238 | | 585 | |

Component

## pH 7.5

Fluorescence

# Stop Vio

Component

| 1 | 2 | 3 |
| --- | --- | --- |

## pH 5.0

Fluorescence

| 1 | 2 | 3 |
| --- | --- | --- |

| Lifetime state | 1 | 2 | 1 | 2 | 1 | 2 |
| --- | --- | --- | --- | --- | --- | --- |
| Lifetime | 0.49 | 2.27 | 0.49 | 2.27 | 0.49 | 2.27 |
| Intensity | 0.052 | 0.11 | 0.098 | 0.47 | 0.14 | 0.085 |

| Lifetime state | 1 | 2 | 1 | 2 | 1 | 2 |
| --- | --- | --- | --- | --- | --- | --- |
| Lifetime | 0.53 | 2.20 | 0.53 | 2.45 | 0.53 | 2.20 |
| Intensity | 0.12 | 0.096 | 0.048 | 0.33 | 0.12 | 0.071 |

Transition rates Transition rates

| Lifetime state Final \ Initial | 1 | 2 | 1 | 2 | 1 | 2 |
| --- | --- | --- | --- | --- | --- | --- |
| 1 | 0.28 | 6.0 | 0.17 | 1500 | 0.047 | 0.0014 |
| 2 | 13 | 0.26 | 320 | 0.25 | < 0.001 | 0.027 |
| Population | 0.32 | 0.68 | 0.82 | 0.18 | 0.96 | 0.040 |
| Free energy  diﬀerence | -156 | | 315 | | 695 | |

| Lifetime state Final \ Initial | 1 | 2 | 1 | 2 | 1 | 2 |
| --- | --- | --- | --- | --- | --- | --- |
| 1 | 0.23 | 5.2 | 0.12 | 870 | 0.10 | 0.0010 |
| 2 | 9.7 | 0.20 | 280 | 0.16 | < 0.001 | 0.069 |
| Population | 0.35 | 0.65 | 0.75 | 0.25 | 0.95 | 0.050 |
| Free energy  diﬀerence | -129 | | 233 | | 649 | |

Component

## pH 7.5

Fluorescence

# WT Zea

Component

| 1 | 2 | 3 |
| --- | --- | --- |

## pH 5.0

Fluorescence

| 1 | 2 | 3 |
| --- | --- | --- |

| Lifetime state | 1 | 2 | 1 | 2 | 1 | 2 |
| --- | --- | --- | --- | --- | --- | --- |
| Lifetime | 0.47 | 2.35 | 0.47 | 2.35 | 0.47 | 2.35 |
| Intensity | 0.11 | 0.022 | 0.0076 | 1.0 | 0.091 | 0.23 |

| Lifetime state | 1 | 2 | 1 | 2 | 1 | 2 |
| --- | --- | --- | --- | --- | --- | --- |
| Lifetime | 0.44 | 1.97 | 0.44 | 1.97 | 0.44 | 1.97 |
| Intensity | 0.0073 | 0.13 | 0.060 | 0.41 | 0.046 | 0.020 |

Transition rates Transition rates

| Lifetime state Final \ Initial | 1 | 2 | 1 | 2 | 1 | 2 |
| --- | --- | --- | --- | --- | --- | --- |
| 1 | 0.56 18 | | 0.038 730 | | 0.021 | < 0.001 |
| 2 | 41 0.56 | | 12 0.038 | | < 0.001 | 0.021 |
| Population | 0.31 | 0.69 | 0.98 | 0.020 | 0.42 | 0.58 |
| Free energy  diﬀerence | -166 | | 857 | | -66 | |

| Lifetime state Final \ Initial | 1 | 2 | 1 | 2 | 1 | 2 |
| --- | --- | --- | --- | --- | --- | --- |
| 1 | 1.2 | 130 | 0.14 | 860 | 0.11 | 0.0031 |
| 2 | 25 | 0.33 | 53 | 0.23 | < 0.001 | 0.11 |
| Population | 0.97 | 0.030 | 0.84 | 0.16 | 0.94 | 0.060 |
| Free energy  diﬀerence | 342 | | 581 | | 712 | |

Component

## pH 7.5

Fluorescence

# Stop Zea

Component

| 1 | 2 | 3 |
| --- | --- | --- |

## pH 5.0

Fluorescence

| 1 | 2 | 3 |
| --- | --- | --- |

| Lifetime state | 1 | 2 | 1 | 2 | 1 | 2 |
| --- | --- | --- | --- | --- | --- | --- |
| Lifetime | 0.46 | 2.17 | 0.46 | 2.17 | 0.46 | 2.17 |
| Intensity | 0.051 | 0.066 | 0.078 | 0.28 | 0.11 | 0.037 |

| Lifetime state | 1 | 2 | 1 | 2 | 1 | 2 |
| --- | --- | --- | --- | --- | --- | --- |
| Lifetime | 0.46 | 1.90 | 0.46 | 1.90 | 0.46 | 1.90 |
| Intensity | 0.060 | 0.11 | 0.043 | 0.14 | 0.11 | 0.083 |

Transition rates Transition rates

| Lifetime state Final \ Initial | 1 | 2 | 1 | 2 | 1 | 2 |
| --- | --- | --- | --- | --- | --- | --- |
| 1 | 0.29 | 6.7 | 0.15 | 1900 | 0.068 | 0.0017 |
| 2 | 9.9 | 0.29 | 270 | 0.15 | < 0.001 | 0.066 |
| Population | 0.40 | 0.60 | 0.87 | 0.13 | 0.97 | 0.030 |
| Free energy  diﬀerence | -82 | | 400 | | 768 | |

| Lifetime state Final \ Initial | 1 | 2 | 1 | 2 | 1 | 2 |
| --- | --- | --- | --- | --- | --- | --- |
| 1 | < 0.001 | 0.56 | 1.7 | 470 | 0.050 | 0.0011 |
| 2 | 0.97 | 0.25 | 67 | 1.2 | < 0.001 | 0.041 |
| Population | 0.37 | 0.63 | 0.88 | 0.12 | 0.90 | 0.010 |
| Free energy  diﬀerence | -114 | | 406 | | 466 | |
