# Supplementary material for: Identification of distinct pH- and zeaxanthin-dependent quenching in LHCSR3 from Chlamydomonas reinhardtii
Source: eLife. 2021 Jan 15;10:e60383. doi: 10.7554/eLife.60383 (PMC7864637; doi:10.7554/eLife.60383)
Supplement: Figure 3—source data 3. — Kinetics of Figure 3—figure supplement 5—source data 1 were fitted with a two-exponential decay function using Vinci two software from ISS. Fractions (fi) and time constants (τi) are reported. Average fluorescence lifetimes were calculated as Σfiτi. Errors are reported as standard deviation (n = 2). [file elife-60383-fig3-data3.docx]

|  | τ1 (ns) | f1 | τ2 (ns) | f2 | τavg (ns) |
| --- | --- | --- | --- | --- | --- |
| CC4349 t0 b2 | 3.560 ± 0.014 | 0.926 ± 0.013 | 0.438 ± 0.049 | 0.074 ± 0.013 | 3.327 ± 0.058 |
| CC4349 t60 b2 | 3.505 ± 0.021 | 0.904 ± 0.006 | 0.629 ± 0.091 | 0.096 ± 0.006 | 3.230 ± 0.012 |
| zep B2 | 3.457 ± 0.021 | 0.811 ± 0.007 | 0.920 ± 0.057 | 0.189 ± 0.007 | 2.992 ± 0.045 |
| CC4349 t0 b3 | 3.410 ± 0.099 | 0.961 ± 0.000 | 0.506 ± 0.107 | 0.039 ± 0.000 | 3.296 ± 0.100 |
| CC4349 t60 b3 | 3.345 ± 0.049 | 0.963 ± 0.003 | 1.825 ± 0.049 | 0.037 ± 0.003 | 3.289 ± 0.041 |
| zep B3 | 3.280 ± 0.000 | 0.843 ± 0.012 | 1.000 ± 0.028 | 0.157 ± 0.012 | 2.921 ± 0.032 |
